# Simple, Single-Shot Phosphoproteomic Analysis of Heat-Stable Tau Identifies Age-Related Changes in pS235- and pS396-Tau Levels in Non-human Primates

**DOI:** 10.3389/fnagi.2021.767322

**Published:** 2021-11-18

**Authors:** Shannon N. Leslie, Jean Kanyo, Dibyadeep Datta, Rashaun S. Wilson, Caroline Zeiss, Alvaro Duque, TuKiet T. Lam, Amy F. T. Arnsten, Angus C. Nairn

**Affiliations:** ^1^Department of Psychiatry, Yale School of Medicine, Yale University, New Haven, CT, United States; ^2^Interdepartmental Neuroscience Program, Yale School of Medicine, Yale University, New Haven, CT, United States; ^3^Keck MS & Proteomics Resource, Yale School of Medicine, Yale University, New Haven, CT, United States; ^4^Department of Neuroscience, Yale School of Medicine, Yale University, New Haven, CT, United States; ^5^Department of Comparative Medicine, Yale School of Medicine, Yale University, New Haven, CT, United States; ^6^Department of Molecular Biophysics and Biochemistry, Yale University, New Haven, CT, United States

**Keywords:** tau, phosphoproteomics, aging, monkey, Alzheimer’s disease

## Abstract

Age is the most significant risk factor for Alzheimer’s disease (AD), and understanding its role in specific aspects of AD pathology will be critical for therapeutic development. Neurofibrillary tangles composed of hyperphosphorylated tau are a quintessential hallmark of AD. To study age-related changes in tau phosphorylation, we developed a simple, antibody-free approach for single shot analysis of tau phosphorylation across the entire protein by liquid-chromatography tandem mass spectrometry. This methodology is species independent; thus, while initially developed in a rodent model, we utilized this technique to analyze 36 phosphorylation sites on rhesus monkey tau from the prefrontal cortex (PFC), a region vulnerable to AD-linked degeneration. Data are available via ProteomeXchange with identifier PXD027971. We identified novel, age-related changes in tau phosphorylation in the rhesus monkey PFC and analyzed patterns of phosphorylation change across domains of the protein. We confirmed a significant increase and positive correlation with age of phosphorylated serine 235 tau and phosphorylated serine 396 tau levels in an expanded cohort of 14 monkeys. Histology showed robust labeling for tau phosphorylated at these sites in vulnerable layer III pyramidal cells in the PFC. The results presented in this study suggest an important role of the natural aging process in tau phosphorylation in rhesus monkey.

## Introduction

Alzheimer’s disease (AD) represents a major public health crisis worldwide. The vast majority of AD cases are sporadic. Age is the most significant known risk factor for the disease, with an individual’s risk of developing AD more than doubling every decade between 65 and 85 years (Alzheimer’s Association, 2021). Given the clear risk that aging presents, it is critical that we understand the role it plays in AD development.

**FIGURE 1:**
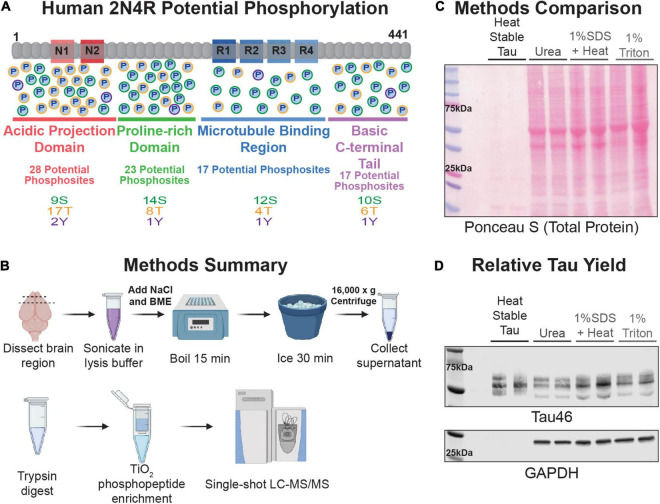
Technique Overview. **(A)** Schematic representation of the longest isoform of human tau, 2N4R. The domains are represented and underlined with a length proportional to their amino acid number in the protein (acidic projection domain is red, proline-rich region is green, microtubule binding region is blue, and the basic C-terminal tail is lavender). Potential phosphorylation sites are represented by circles with a “P” in the middle in each domain and are color coded by residue type (serine outlined in green, threonine in orange, tyrosine in purple). **(B)** Cartoon summary of the heat-stable tau enrichment technique and mass spectrometry work-flow described in this study. **(C)** Samples were separated by SDS-PAGE and proteins transferred to nitrocellulose. Image of the ponceau S-stained nitrocellulose membrane of various rat FC lysates. Lysate prepared from two independent rat FC tissue samples were run side-by-side for each buffer type as labeled at the top of the image. An amount relative to starting mass was loaded for each sample. This was equivalent to 4 μL for the heat-stable tau, urea, and 1% SDS + heat and 10 μL for the 1% triton samples. **(D)** Immunoblot of the membrane shown in **(C)** blotted with antibodies against total tau (Tau46) and GAPDH.

One of the key pathologic hallmarks of AD are neurofibrillary tangles (NFTs) composed of hyperphosphorylated tau. NFTs are well correlated with the progression and cognitive symptoms of the disease (Nelson et al., 2012). The primary constituent of NFTs, tau, is a microtubule-associated protein that binds and stabilizes microtubules (Murphy et al., 1977; Kutter et al., 2016; Barbier et al., 2019). Tau is expressed in six isoforms that are distinguished by the inclusion of near-amino-terminal inserts (0N, 1N, 2N) and carboxy-terminal repeats (3R or 4R) (Wang and Mandelkow, 2016). Tau can be modified by a variety of post-translational modifications (PTMs) including phosphorylation, acetylation, and ubiquitination. Phosphorylation is the most abundant PTM. The 2N4R human tau isoform is 441 amino acids long and there are 85 potential phosphorylation sites (Figure 1A). Phosphorylation of tau plays an important role in modulating the protein’s normal physiology; notably, decreasing tau’s binding and stabilizing of microtubules (Mandelkow et al., 1995; Barbier et al., 2019). However, abnormal tau phosphorylation has long been recognized for its proposed role in AD pathology (Kimura et al., 1996; Augustinack et al., 2002; Avila, 2006). Tau from AD patients has 3–4 fold higher levels of phosphorylation than demographically matched, cognitively unimpaired individuals (Gong et al., 2005). Normally tau is a highly soluble, intrinsically disordered protein, but following modification, in a manner which is yet to be fully understood, it aggregates and becomes insoluble (Wang and Mandelkow, 2016; Wegmann et al., 2021). Higher molecular weight tau species, presumably more highly modified, are associated with a propensity to trigger tau aggregation (Dujardin et al., 2020). Recent research suggests the pattern of tau phosphorylation, rather than levels at individual phosphorylation sites, may play a critical role in the heterogeneity observed in clinical symptoms of AD patients (Dujardin et al., 2020; Wesseling et al., 2020). Thus, it is important to consider the full spectrum of tau phosphorylation with age to better understand what role age-related molecular changes play in susceptibility to tau pathology.

Non-human primate models provide an important intermediate for the study of tau pathology but are more limited in the molecular tools and sample sizes available. The cortico-cortical connections vulnerable to AD pathology are the same circuits that are most expanded in human evolution making more closely related non-human primates invaluable models of disease (Rakic, 2009). For example, rhesus macaques naturally develop amyloid and tau pathology at advanced ages making them invaluable models for sporadic AD pathology (Paspalas et al., 2018). Importantly, the 2N4R tau isoform from macaques shares 98% sequence similarity with the human protein and contains 83 potential phosphorylation sites. However, unlike human tissue, monkey tissue can be collected with extremely short (on the order of minutes) post-mortem intervals (PMI), which reduces the loss of potential phosphorylation that can be caused by phosphatase activity during the PMI (Matsuo et al., 1994; Li et al., 2003; Wang et al., 2015). Nonetheless, there are still obstacles to studying tau in both humans and macaques, including limited sample sizes, variable, often unknown, medical histories, and more limited opportunities for mechanistic studies compared to rodent models. Analysis of tau across patient samples and various disease models is important; therefore, designing techniques that are easily applied across species is useful.

Proteomics approaches enable a broader and more unbiased view of PTMs on tau. A variety of mass spectrometry (MS) approaches have been used to analyze tau and its modifications, ranging from intact protein (i.e., top-down) native MS approaches (Nshanian et al., 2019; Drepper et al., 2020) to peptide-level (i.e., bottom-up), targeted parallel-reaction monitoring (PRM) MS approaches (Mair et al., 2016; Barthélemy et al., 2019). These analyses have led to critical insights, including the identification of a potential novel biomarker for the diagnosis of AD, pT217-tau (Barthélemy et al., 2020a,b; Janelidze et al., 2020; Palmqvist et al., 2020). When determining the best approach for proteomic analysis of tau there are several important considerations, including the pool of tau the assay will capture, the breadth of PTMs captured by the assay, and the ease of implementation.

We sought to develop a simple, easy to implement approach, for single-shot analysis of tau phosphorylation that could be adapted to various experimental designs, particularly cross-species. In order to enrich for a broad pool of tau in a species non-specific fashion, we utilized a heat-stable approach. We used a standard proteomic work-flow to analyze the samples including trypsin digestion followed by titanium dioxide (TiO_2_) phosphopeptide enrichment and analysis via liquid-chromatography tandem MS (LC-MS/MS). This technique was optimized in aging rodents but was transitioned, without modification, to macaques. To study age-related changes in tau phosphorylation that may correlate with AD tau pathology risk, we studied monkey dorsolateral prefrontal cortex (dlPFC) from both young and aged animals. We selected animals at advanced ages where we previously observed phosphorylation changes (Paspalas et al., 2018; Datta et al., 2020a) and cognitive impairments (Rapp and Amaral, 1989; Wang et al., 2011). We chose the dlPFC as it is a region of the brain especially vulnerable to AD tau pathology (Braak and Braak, 1991) and it plays a critical role in cognitive functions that are susceptible to age-related decline as well as deficits in AD (Wang et al., 2011; Arnsten et al., 2012). We analyzed changes in 36 identified phosphosites from the samples enriched using TiO_2_. We identified phosphosites that changed with age in the dlPFC region of macaques and followed-up the identification of elevated phosphorylation at serine 235 (pS235-tau) and serine 396 tau (pS396-tau) in additional animals. We found a significant positive correlation of phosphorylation at both sites with advancing age and an overall elevation in aged animals compared to young in an expanded cohort of 14 macaques. These results were further supported by robust labeling of layer III and layer V/VI dlPFC pyramidal neurons for pS235-tau and pS396-tau in aged macaques. These results demonstrate the flexibility, ease, and impact of this approach to further study age-related changes in tau phosphorylation.

## Methods

### Animals

Animals were cared for in accordance with the guidelines of Yale University Institutional Animal Care and Use Committee, and Public Health Service requirements as described in the Guide for the Care and Use of Laboratory Animals. Yale University is accredited by the American Association for Accreditation of Laboratory Animal Care (AAALAC). Further detail on animal care and handling is included in the Supplementary Material.

Rodent tissue was collected from 3.5-month-old young and 30-month-old Sprague Dawley aged rats. Previous analysis of 3 of the 4 aged animals and all of the young animals has been published along with a description of the tissue collection (Leslie et al., 2020). Briefly, the frontal cortex block of the tissue was dissected with a razor blade and tissue was immediately frozen on dry ice. All rats were male.

Macaque tissue was collected between May 2015 and January 2020. Tissue was collected as described in Datta et al. (2021) and was frozen rapidly in liquid nitrogen. PMI was minimized as much as possible but ranged from 10 min to approximately 1 h. The macaque cohort was all female except for the 7-year-old animal, who was male. Nine of the 14 animals utilized in this study were also characterized in Datta et al. (2020b, 2021). The animal labeled as “low” is the 19.5-year-old animal highlighted previously (Datta et al., 2021). Animals were divided between young (<18 years) and aged (>18 years) using a standard age cut-off that is utilized by other researchers (Bartus et al., 1978). Further information is included in the Supplementary Material.

### Western Blots

Samples were prepared in the various lysis buffers which are described in the Supplementary Material and listed in Figure 1 legend. Samples were run on tris-glycine gels and transferred onto nitrocellulose membranes. Antibodies used for biochemical analysis were Total Tau46 (CST 4019, 1:1000), pS235-tau (Abcam ab133253, 1:1000), pS396-tau (Thermo 44-752G, 1:1000), pT181-tau (CST 12885S 1:1000), GAPDH (Millipore CB1001-500, 1:10,000). Blots were developed on a LI-COR Odyssey scanner. Quantification was done in Image Studio Lite with background subtracted. All protein levels were normalized to glyceraldehyde 3-phosphate dehydrogenase (GAPDH) for quantification. Statistical analyses were performed in GraphPad Prism. The appropriate test was chosen based on the distribution of the values and is listed in the figure legends for either a comparison of means or correlation with age. Further details are included in the Supplementary Material.

### Heat-Stable Preparation

Small sections of tissue were kept on dry ice while they were carefully cut with a razor blade and weighed for lysis preparation. Lysis buffer (Tris buffered solution detailed in Supplementary Material) was added to each sample at a ratio of 4 μl lysis buffer for every 1 mg of tissue. Samples were briefly sonicated following addition of lysis buffer. NaCl (22 μl/mL 5 M) and beta-mercaptoethanol (BME; 55 μl/mL) were added and samples were briefly vortexed at maximum speed. Samples were boiled for 15 min at 100°C and vortexed again. Samples were cooled on ice for 30 min, then centrifuged at 4°C, 16,000 × *g* for 10 min. Supernatant was collected for subsequent analysis.

### Mass Spectrometry Sample Preparation

Samples were diluted with 100 mM ammonium bicarbonate (ABC) equivalent to half final volume minus the reduction and alkylation volumes to ensure the BME was diluted. Either 45 mM dithiothreitol (DTT) or 100 mM Tris(2-carboxyethyl)phosphine (TCEP) was added as the reducing agent at a 1:10 ratio and incubated at 37°C for 30 min. Then samples were alkylated with a 1:10 dilution of 100 mM iodoacetamide (IAA) for DTT reduced samples, or 150 mM IAA for TCEP reduced samples, at room temperature in the dark for 30 min. Following reduction and alkylation all samples were digested with 1 μg trypsin per 20 μL lysate overnight at 37°C. Following digestion, samples were acidified with 20% trifluoroacetic acid (TFA) and de-salted using Nest Group C18 macrospin columns (HMMS18V) following the manufacturer’s instructions. Eluent from the C18 columns was dried in a SpeedVac and analyzed as the total lysate. Phosphopeptides were enriched using TopTip TiO_2_ columns (Part No. TT1TIO). For TiO_2_ enrichment dried peptides from the de-salt were brought up in 50 μL of 70 mM L-glutamic acid, 65% acetonitrile, 2% TFA in water. Samples were then enriched on the TiO_2_ columns following the manufacturer’s instructions. Eluent was dried in a SpeedVac.

### Mass Spectrometry

Samples were analyzed using a Q-Exactive Plus (Thermo Fisher Scientific) LC MS/MS system equipped with a Waters nanoACQUITY UPLC system, that used a Waters Symmetry C_18_ 180 μm × 20 mm trap column and a 1.7 μm (75 μm × 250 mm) nanoACQUITY UPLC column (37°C) for peptide separation. Trapping was done at 5 μl/min, 99% Buffer A for 3 min. Peptide separation was performed with a linear gradient over 140 min at a flow rate of 300 nL/min. Precursor mass scans [300–1,700 m/z range, Automatic Gain Control (AGC) target value of 3E6, maximum ion injection times 45 ms] were acquired and followed by HCD based fragmentation (normalized collision energy 28). A resolution of 70,000 at m/z 200 was used for MS1 scans, and up to 20 dynamically chosen, most abundant, precursor ions were fragmented (isolation window 1.7 m/z). The tandem MS/MS scans were acquired at a resolution of 17,500 at m/z 200 (AGC target value of 1E5, maximum ion injection times 100 ms). Further details are in the Supplementary Material.

### Data Analysis

Spectra were searched on Mascot (Perkins et al., 1999) (Matrix Science Inc., Boston, MS, United States) version 2.7.0 in Proteome Discoverer 2.2.0.388 with a fragment tolerance of 0.020 Da (monoisotopic), parent tolerance of 10.00 PPM (monoisotopic), no fixed modifications, and variable modifications as listed in the Supplementary Material. Additionally, data were analyzed using Scaffold and Scaffold PTM (Proteome Software, Portland, OR, United States). Scaffold PTM was used to annotate PTM sites derived from MS/MS sequencing results obtained using Scaffold (version Scaffold_4.11.1), using a site localization algorithm developed by Beausoleil et al. (2006). Further details are presented in the Supplementary Material. The mass spectrometry proteomics data have been deposited to the ProteomeXchange Consortium via the PRIDE (Perez-Riverol et al., 2019) partner repository with the dataset identifier PXD02797.

Phosphorylation abundance was calculated using spectral counts. Spectral counts of phosphopeptides were normalized by total spectral accounts assigned to 2N4R tau. This was compared to total ion current (TIC) results in the Supplementary Material. Correlation between phosphosites was run in R Studio using cor (Pearson correlation) to analyze the data and corrplot to represent the data. Sites for validation were chosen if the two aged macaques had a higher normalized phosphorylation than the two young macaques. Rodent data was analyzed with a 2-way ANOVA as described in the figure legends using GraphPad Prism.

### Single-Label Immunoperoxidase Immunohistochemistry

For pS235-tau and pS396-tau immunolabeling, two female aged rhesus macaques (24 and 31 years old) were used. Animal procedures including anesthesia, perfusions, and histological processing are described in the Supplementary Material.

For single-label immunoperoxidase immunohistochemistry, sections of dlPFC were transferred for 1 h to Tris-buffered saline (TBS) containing 5% bovine serum albumin, plus 0.05% Triton X-100 to block non-specific reactivity, and incubated in antibodies for pS235-tau or pS396-tau (same antibodies as used for biochemistry) in TBS for 48 h at 4°C. The tissue sections were incubated in goat anti-rabbit biotinylated antibody (Vector Laboratories) at 1:300 in TBS for 2 h, and developed using the Elite ABC kit (Vector Laboratories) and diaminobenzidine (DAB) as a chromogen. Omission of the primary antibody eliminated all labeling. Sections were mounted on microscope slides and dlPFC layer III and layer V/VI was photographed under an Olympus BX51 microscope equipped with a Zeiss AxioCam CCD camera. Zeiss AxioVision imaging software was used for imaging and data acquisition.

### Graphics

Cartoon depiction of the technique was created with BioRender.com. Graphs and heatmaps were made on GraphPad Prism. The correlation matrix was made in R Studio.

## Results

Utilizing rat frontal cortex (FC) blocks, tau was enriched using a beta-mercaptoethanol and high salt heat-stable approach (Figure 1B). This approach yielded a notably lower overall protein concentration compared to a standard proteomics 8 M Urea lysis, a SDS heat stable enrichment, or a detergent soluble 1% triton lysis (Figure 1C). Despite a lower total protein yield, the heat-stable samples had the same or greater yield of tau without the presence of cytosolic proteins like GAPDH (Figure 1D). Following either DTT or TCEP reduction, tryptic protein digestion, and TiO_2_ phosphopeptide enrichment, robust coverage of the tau protein and identification of ∼30 phosphosites was achieved per sample in young and aged wildtype rodents (*N* = 4, 4) (Supplementary Figure 1 and Supplementary Table 1). Although the DTT reduction approach yielded some unique phosphosite identifications, all subsequent data presented utilized TCEP reduction, which produced higher spectral counts, greater total coverage, and higher identification of phosphosites (Supplementary Figures 1A,B). In rodents, there was no significant effect of age on levels of total phosphorylation. Overall, there was only a slight elevation in mean phosphorylation in aged animals (mean young = 0.0198, mean aged = 0.0208, standard error of difference = 0.0018) that was non-significant (Supplementary Figure 1C). Given previous data from rats, where large cohorts were required to detect small changes in phosphorylation by traditional biochemical methods this was not a surprising result (Leslie et al., 2020). Nonetheless, these results demonstrated a broad analysis of tau was possible using this approach, even in an animal model exhibiting a relatively low level of tau phosphorylation.

**FIGURE 2:**
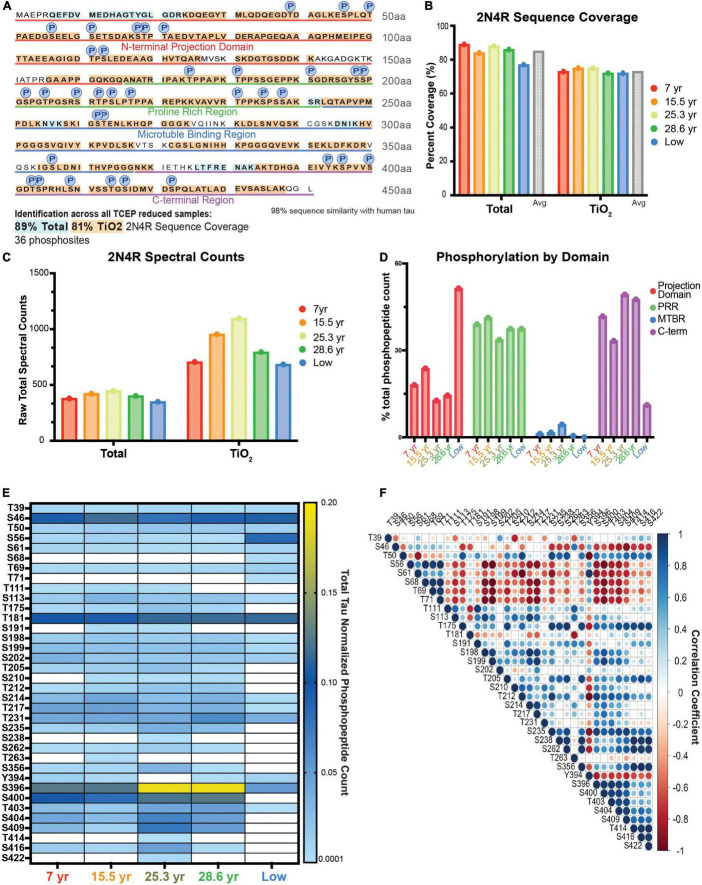
Analysis of age-related changes in monkey heat stable tau via single-shot LC-MS. **(A)** Coverage map of 2N4R isoform of tau from all TCEP reduced samples (ten samples total, five unenriched samples, five TiO_2_ enriched samples). Amino acids represented in the total, unenriched lysate only, are highlighted in blue. Those identified in both unenriched and TiO_2_ enriched samples are highlighted in orange. 36 unique phosphosites were detected and are identified by a circle labeled with a P directly above the phosphorylated residue. **(B–E)** Animals are arranged in age order, except “low” is farthest right. **(B)** Bar graph of the percent coverage of the 2N4R isoform of tau achieved in each LC-MS run. Runs are color coded by the age of the animal from which the sample was derived as indicated in the key on the right. There is a total unenriched and TiO_2_ enriched group. The average for each group is plotted by a gray bar at the right-hand side of the group. **(C)** Total spectral counts of the 2N4R isoform of tau detected in each run. Runs are color coded by the age of the animal from which the sample was derived as indicated in the key on the right. There is a total unenriched and TiO_2_ enriched group. **(D)** Bar graph representation of proportion of phosphorylation from each domain. The total spectral counts representing phosphorylated residues in each domain were added together and divided by the total phosphopeptide spectral counts for that animal. Results are presented as a percent. The graph is color-coded to match the domain identification in **(A)** and Figure 1A. Each run is labeled by the animal identification on the *x*-axis. **(E)** Heat map representation of normalized spectral count phosphorylation values from each animal. Columns are identified by the animal’s age in years or by the “low” designation described in the text. The heat map ranges from the minimum possible value given any spectra were identified, 0.0001, in light blue to dark blue at the mid-point of values, 0.01, to yellow for the maximum value, 0.20, as indicated in the key on the right. When there were no spectral counts, the boxes are white. Values are the ratio of phosphopeptide spectral counts for the assigned residue over total tau 2N4R spectral counts in that sample run. **(F)** Correlation matrix representing the relationship between different phosphorylation sites based on the values shown in **(E)**. A perfect positive correlation is represented by a large dark blue circle and a perfect inverse correlation is shown by a large dark red circle as indicated by the key on the right.

We moved forward with analysis of dlPFC tissue from a cohort of 5 macaques that included two young animals and two aged. As a validation of our approach, we also included a fifth 19.5-year-old animal (termed “low”) shown by western blot to have notably low levels of phosphorylation in comparison to our entire macaque cohort in a previous study (Datta et al., 2021). Utilizing the heat-stable approach, we achieved a total of 89% (average 85%) coverage of 2N4R tau in unenriched, TCEP reduced samples and a total of 81% coverage (average 73%) in TiO_2_ enriched samples (Figures 2A,B). In addition to tau, we identified over 1,000 other proteins, including other known heat-stable proteins, such as alpha-synuclein (Supplementary Table 2 “Scaff_Protein_Report”). We detected and quantified 36 phosphosites across all domains of tau (Figure 2A), and achieved similar coverage and total spectral counts (Figures 2B,C). Analysis of the domain distribution of phosphopeptides showed that young animals had the highest proportion of phosphopeptides in the proline rich region (PRR), while aged animals had the highest level of phosphorylation in the C-terminal region (C-term). Notably, the “low” animal had the highest proportion of phosphorylation in the projection domain (Figure 2D). All animals had the lowest spectral count of phosphopeptides within the microtubule binding region (MTBR; Figure 2D). Overall, the phosphoproteomics results captured the patterns of phosphorylation expected based on previous traditional biochemical analyses. The “low” animal had lower levels of overall phosphorylation consistent with previous results (Figure 2E “low”; Datta et al., 2021). Additionally, the older animals had higher overall levels of phosphorylation, as expected from previous results (Paspalas et al., 2018; Datta et al., 2021).

A few individual sites stood out as more highly elevated in the two aged animals compared to young. These results were comparable whether we analyzed via spectral counts or total ion current (TIC; Supplementary Figure 2). Serine 396 is a striking example in which the aged animals had a higher degree of phosphorylation (yellow) compared to the young animals (light blue) (Figure 2E). Other changes, such as serine 235, were consistently elevated in the 2 aged animals versus the 2 young but were not as striking as serine 396 in effect size. Notably, not all phosphorylation sites were elevated in aged animals. For example, serine 56 was relatively more highly phosphorylated in the animal with “low” total level of phosphorylation and higher in both young animals than either aged animal (Figure 2E). Thus, we were interested in how phosphorylation levels at different residues correlated with each other. We found good correlation between most sites that were in close proximity to one another, but anti-correlation between many sites in the projection domain relative to many in the C-terminal domain (Figure 2F). One of the most striking results was an anti-correlation between most phosphorylation sites and both serine 56 and tyrosine 394 (Figure 2F).

**FIGURE 3:**
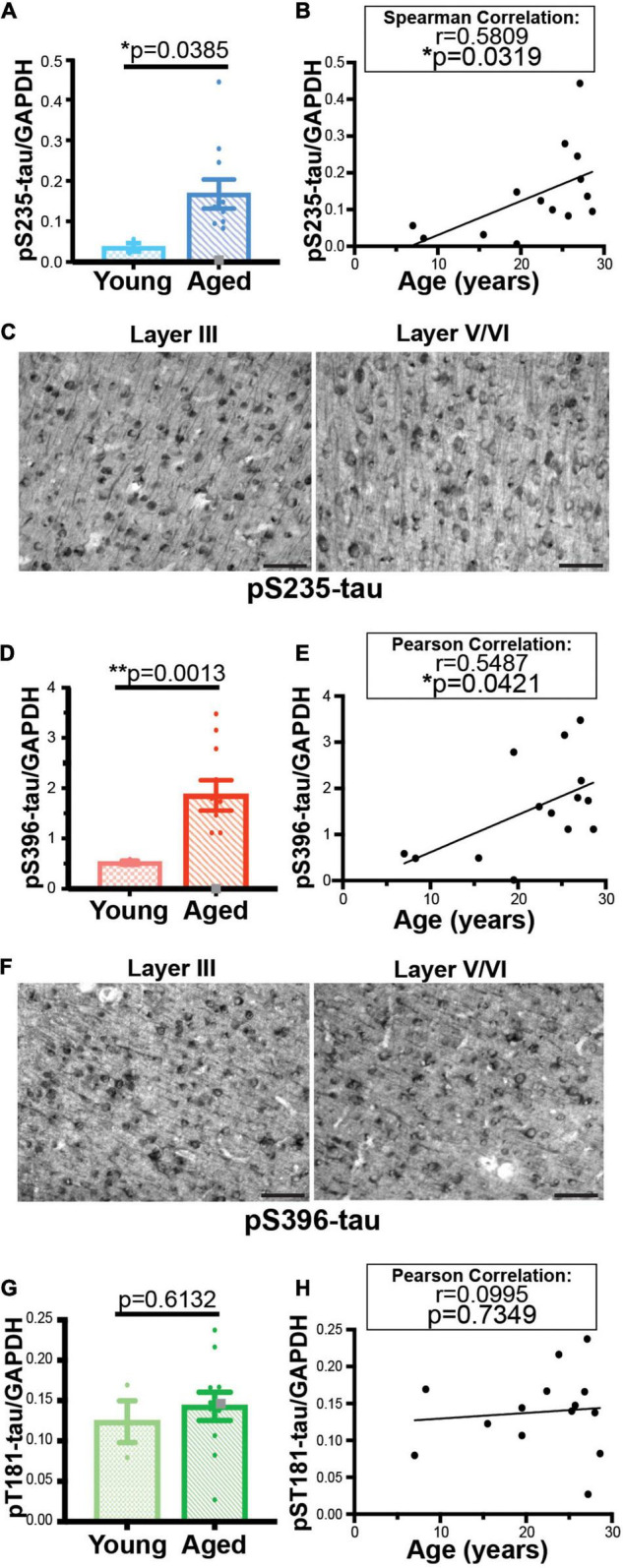
Age-related changes in monkey S235 and S396 validate mass spectrometry results. The 19.5-year-old animal denoted as “low” in the proteomics study and western blots is indicated by a gray square in the graphs. **(A)** Bar graph summarizing a means comparison of pS235-tau between young (light blue, *N* = 3) and aged (dark blue, *N* = 11) macaques. Individual data points are plotted for each group. Due to a lack of normal distribution of pS235-tau with age the means were compared using a Mann–Whitney test (*p = 0.0385). **(B)** Quantification of pS235-tau normalized by GAPDH is plotted by age. Samples were not normally distributed so the correlation coefficient was computed using a Spearman correlation (*r* = 0.5809, *p = 0.0319) but a standard linear regression is represented on the graph. **(C)** Immunohistochemistry revealed robust immunolabeling for pS235-tau in aged macaque dlPFC layer III and layer V/VI, particularly in pyramidal neurons along apical dendrites and in the cell body. Scale bar, 50 μm. **(D)** A bar graph summarizing a means comparison of pS396-tau between young (light pink, *N* = 3) and aged (red, *N* = 11) macaques. Individual data points are plotted for each group. Given the normal distribution of pS235-tau with age but unequal variance, the means were compared using a Welch’s *t*-test (**p = 0.0013). **(E)** Quantification of pS396-tau normalized by GAPDH is plotted by age. Samples were normally distributed so the correlation coefficient was computed using a Pearson correlation (*r* = 0.5487, *p = 0.0412) and a standard linear regression is represented on the graph. **(F)** Immunohistochemistry identified prominent immunolabeling for pS396-tau in aged macaque dlPFC layer III and layer V/VI, especially within the perisomatic compartment and along apical dendrites in pyramidal neurons. Scale bar, 50 μm. **(G)** A bar graph summarizing a means comparison of pT181-tau between young (light green, *N* = 3) and aged (dark green, *N* = 11) macaques. Individual data points are plotted for each group. Given the normal distribution of pT181-tau with age but unequal variance, the means were compared using an unpaired *T*-Test (*p* = 0.6132). **(H)** Quantification of pT181-tau normalized by GAPDH is plotted by age. Samples were normally distributed so the correlation coefficient was computed using a Pearson correlation (*r* = 0.0995, *p* = 0.7349) and a standard linear regression is represented on the graph.

We used traditional biochemical measures and histology to validate age-related changes detected in two tau phosphorylation sites (S235 and S396). Analysis of an expanded 14 animal cohort of young and aged macaques confirmed an age-related elevation of both pS235-tau and pS396-tau levels (Figure 3 and Supplementary Figure 3). We found a significant elevation in levels of triton-soluble pS235-tau in aged animals compared to young animals (**p = 0.0046) and a significant correlation with age overall (*p = 0.0319) despite variability in the cohort (Figures 3A,B). Similarly, analysis of dlPFC triton-soluble lysate demonstrated an age-related increase in pS396-tau in aged animals in comparison to young animals (**p = 0.0013) and a significant positive linear correlation with age (*p = 0.0412) (Figures 3D,E). The “low” animal is a notable outlier from the other aged animals. We used immunohistochemistry to localize pS235-tau and pS396-tau in aged macaque dlPFC. We found robust immunolabeling for pS235-tau (Figure 3C and Supplementary Figure 4A) and pS396-tau (Figure 3F and Supplementary Figure 4B) phosphorylation sites across the cortical neuropil, including robust labeling in pyramidal neurons in supragranular dlPFC layer III and infragranular dlPFC layer V/VI. Immunolabeling was visualized in perisomatic compartments and along apical dendrites in pyramidal neurons, including punctate labeling in the neuropil. Changes were not observed in all phosphorylation sites. Similar to what was observed in the proteomic results there were no changes in pT181-tau levels between young and aged animals and no correlation with age (Figures 3G,H and Supplementary Figure 3C). Additionally, the “low” phosphorylation animal had relatively high levels of T181 phosphorylation by western blot similar to what was observed by proteomic analysis.

## Discussion

The results of this study demonstrate a single-shot phosphoproteomic approach for the analysis of tau across species. Utilizing this technique, we were able to analyze cortical tissue from rats and monkeys without any alterations in methodology. The technique identified at least 30 phosphorylation sites on tau in each species. Importantly, we identified and validated two previously unreported age-related changes in tau phosphorylation in macaques, pS235-tau and pS396-tau. Overall, the ability to quickly analyze a broad spectrum of tau phosphorylation appears useful to better understanding the risk that aging plays in AD etiology.

Hyperphosphorylated forms of tau have long been studied for their inclusion in NFTs, a pathological hallmark of AD. However, traditional biochemical techniques do not enable an in-depth analysis of numerous tau phosphorylation sites simultaneously. For this reason, researchers have developed phosphoproteomic approaches to analyze wide swaths of tau phosphorylation in an individual sample (Morris et al., 2015; Mair et al., 2016; Sato et al., 2018; Wesseling et al., 2020). This advancement has already led to important discoveries in human AD; including the identification of a new potential AD biomarker, pT217-tau, in human cerebrospinal fluid (Barthélemy et al., 2020a; Janelidze et al., 2020) and plasma (Palmqvist et al., 2020). While, these methodologies are important for the identification and characterization of AD pathology, and are shedding light on the complexities therein, they often utilize highly optimized protocols. Some methods rely on antibody-based enrichment, which requires the correct epitope be available, soluble, and capable of reliable enrichment in the tissue preparation of interest (Morris et al., 2015; Sato et al., 2018). Others utilized a targeted proteomics approach and heavy labeled peptides to detect tau in various lysates (Mair et al., 2016; Wesseling et al., 2020). These approaches can be challenging to implement across species and study designs without special expertise in this area of mass spectrometry. Thus, we sought to develop a single-shot phosphoproteomic approach for the analysis of tau phosphorylation that could be easily applied to multiple model organisms using standard mass spectrometry protocols often used in discovery proteomics. We chose to use a heat-stable approach for the enrichment of tau, which, rather than relying on the availability or efficacy of a specific epitope, enriches tau based solely on the biochemical properties of the protein. However, it should be noted that aggregated forms of tau including, paired-helical filaments (PHF), still demonstrate resistance to heat-stable enrichments (Jeganathan et al., 2008) and these preparations do not include all species of tau (Miao et al., 2019). Additionally, the TiO_2_-based phosphoenrichment strategy potentially biases the enrichment of singly phosphorylated peptides; however, we did detect doubly and triply phosphorylated tau peptides. Ultimately, we achieved high coverage of tau in our samples irrespective of the species, age, or degree of tau phosphorylation. It should be noted that this method did not detect age-related changes in three phosphorylation sites that we detected previously: pS214-tau, pT231-tau, and pS356-tau (Carlyle et al., 2014; Datta et al., 2021), although we were able to recapitulate the overall low phosphorylation of one of the aged animals (the 19.5-year-old “low” animal). These differences may be due to either lysis conditions, a lack of sensitivity and power, or animal-to-animal variations.

This novel, unbiased approach uncovered two, previously undescribed, age-related changes in monkey tau phosphorylation: there were significant elevations in pS235-tau and pS396-tau in aged animals compared to young, and phosphorylation levels at both residues significantly correlated with age. Phosphorylation at both of these sites has been previously associated with human AD tau pathology (Otvos et al., 1994; Kimura et al., 1996; Brici et al., 2018). Subsequently, there is significant interest in utilizing antibodies directed toward PHF-1 (an antibody that targets pS396/S404-tau) as immunotherapies for AD (Asuni et al., 2007; Bi et al., 2011; Chukwu et al., 2018). However, this is the first report, to our knowledge, of age-related changes in phosphorylation at these two sites in vulnerable regions of the primate brain. A recent publication demonstrated an age-related increase in PHF-1 in synaptic mitochondria of wild-type mice, suggesting this may be a common mechanism of age-related neuronal dysfunction (Torres et al., 2021).

Considering the significant risk of advanced age for AD development, it is important to understand what role aging may play in promoting tau pathology. These animals not only develop tau pathology but also amyloid plaques that are a hallmark of AD. We and others have demonstrated this pathology in animals of similar age to the cohort examined in this study (Mufson et al., 1994; Paspalas et al., 2018; Li et al., 2020). Recent analysis of a subset of our aging monkey cohort revealed age-related elevations in calcium-cAMP-PKA signaling in dlPFC (Datta et al., 2021). This is one potential mechanism linking age-related molecular changes in the dlPFC to increased phosphorylation of S235-tau and S396-tau. Both S235-tau and S396-tau can be phosphorylated by glycogen synthase kinase-3β (GSK3β) (Spittaels et al., 2000; Wang et al., 2002; Lee et al., 2003; Li and Paudel, 2006). Prior phosphorylation of tau by PKA was shown to potentiate GSK3β-dependent phosphorylation at both S235 and S396 (Wang et al., 2002; Liu et al., 2004). The animal with low, overall phosphorylated tau had high levels of the calcium binding protein, calbindin. Calcium has long been implicated in AD (Alzheimer’s Association Calcium Hypothesis Workgroup, 2017) and markers of intracellular calcium dysregulation are related to selective vulnerability and markers of tau pathology (Hof and Morrison, 1991; Datta et al., 2021). Elevated calcium can drive cAMP-PKA production through adenylyl cyclase activation creating a feedforward cycle of calcium-cAMP-PKA activity (Halls and Cooper, 2011). In combination with our previous analysis, the results of this study demonstrate a potentially critical role for regulation of calcium-cAMP-PKA signaling in maintaining proper levels of tau phosphorylation.

Understanding how complex age-related molecular changes impact tau phosphorylation is important for developing novel therapeutic targets for AD. The results of this study suggest that taking a non-biased approach to tau phosphorylation analysis may be beneficial for capturing patterns of tau phosphorylation change that are missed by analysis of individual phosphorylation sites or kinase pathways. A comprehensive view of the tau phosphorylation landscape is critical for better understanding the ways in which aging contributes to AD risk.

## Data Availability Statement

The datasets presented in this study can be found in online repositories. The names of the repository/repositories and accession number(s) can be found in the article. Proteomic data are available via ProteomeXchange with identifier PXD027971.

## Ethics Statement

The animal study was reviewed and approved by the Yale University Institutional Animal Care and Use Committee.

## Author Contributions

SL designed and performed the peptide preparation, analyzed the Scaffold data for proteomics experiments, designed, performed, and analyzed western blot analyses, and wrote the first draft of the manuscript. JK designed and ran the LC-MS/MS analysis of the peptides and advised on methodological decisions for peptide preparation. DD designed, performed, and analyzed immunohistochemistry experiments. RW aided in the design, optimization of the proteomics technique, and helped shape the direction of the project. AD and CZ provided technical expertise and invaluable tissue resources. TL advised and supervised the proteomic assays. AA and AN designed the experiments, supervised the study, and critically revised the manuscript. All authors read, edited, and approved the final manuscript.

## Conflict of Interest

AA and Yale University receive royalties from Shire/Takeda from the United States sales of Intuniv™. They do not receive royalties from international sales or generic Intuniv™. The remaining authors declare that the research was conducted in the absence of any commercial or financial relationships that could be construed as a potential conflict of interest.

## Publisher’s Note

All claims expressed in this article are solely those of the authors and do not necessarily represent those of their affiliated organizations, or those of the publisher, the editors and the reviewers. Any product that may be evaluated in this article, or claim that may be made by its manufacturer, is not guaranteed or endorsed by the publisher.
