# Supplementary material for: Simple, Single-Shot Phosphoproteomic Analysis of Heat-Stable Tau Identifies Age-Related Changes in pS235- and pS396-Tau Levels in Non-human Primates
Source: Front Aging Neurosci. 2021 Nov 18;13:767322. doi: 10.3389/fnagi.2021.767322 (PMC8637411; doi:10.3389/fnagi.2021.767322)
Supplement: Supplementary file 1 [file Data_Sheet_1.docx]

Simple, single-shot phosphoproteomic analysis of heat-stable tau identifies age-related changes in pS235- and pS396-tau levels in non-human primates

Shannon N. Leslie, Jean Kanyo, Dibyadeep Datta, Rashaun S. Wilson, Caroline Zeiss, Alvaro Duque, TuKiet T. Lam, Amy F.T. Arnsten, Angus C. Nairn

**Supplemental Materials**

**Supplemental Methods**

**Animals**

***Rhesus Macaque***

Rhesus macaques were housed and cared for based on NIH guidelines, including pair-housing and daily environmental enrichment. Animals had variable life and medical histories including treatment with long term steroids, incidences of lung mites, kidney failure, etc. Unfortunately, given that these animals are so rare and valuable, cohort sizes remain small relative to what may be observed in other animal studies. Therefore, it is not possible to control for all medical conditions. However, we did not note any obvious associations. We view this cohort as more analogous to human samples with very diverse medical and life histories, not all of which is known or controlled for.

When assessing a potential impact of PMI in our data set, we noted that individual variability was far greater than PMI variability. Two animals with high phosphorylation fell on opposite ends of the PMI window: the 27.1-year-old animal with very high levels of phosphorylation by western blot had one of the longest PMIs (45 min-1 hr) while the 25.3-year-old animal with similarly high levels of phosphorylation had one of the shortest (~15 min). The 7-year-old animal with low relatively levels of phosphorylation also had a short PMI (8.5 min). Given the many years over which animal tissue was collected, PMI windows range from 0.5-15 min, further limiting analysis. Nonetheless, all PMIs are within 1 hr and were far less than what is typically observed with human samples.

***Biochemistry tissue collection***

Rat tissue was collected as described previously (Leslie et al., 2020). One young rat was only used for DTT reduction but not TCEP reduction because that animal did not have sufficient tissue from the front cortex region for both digests. Tissue was stored at -80°C until lysis. All rats were male due to ordering constraints of aged animals.

Macaques were collected between May 2015 and January 2020. Samples were stored at -80°C until analysis. The animal labelled “low” was demonstrated to have low levels of tau phosphorylation at multiple sites and over all low levels of modification as indicated by a lack of band shift in a total tau blot, which were recently published(Datta et al., 2021). Animals were divided between young and aged utilizing an 18 year cut-off that is a standard for the field and utilized by other researchers (Bartus et al., 1978).

***Heat-stable preparation***

Tris lysis buffer included 10 mM Tris-Hydrochloric acid (HCl), 140 mM sodium chloride (NaCl), 2 mM ethylene glycol-bis(β-aminoethyl ether)-N,N,N′,N′-tetraacetic acid (EGTA), PhosStop phosphatase inhibitor (Sigma), cOmplete protease inhibitor cocktail (Sigma). Sonication was done for 20 sec using a Sonicator Ultrasonic Processor XL. Samples were immediately placed on ice and if any visible tissue pieces remained, they were sonicated another 10 sec. Sonication was done at the second to lowest setting.

***Immunohistochemistry***

Macaques were deeply anesthetized prior to transcardial perfusion of PBS, followed by 4% paraformaldehyde/0.05% glutaraldehyde in 100 mM phosphate-buffer (PB). Following perfusion, a craniotomy was performed, and the entire brain was removed and dissected, including a frontal block containing the primary region of interest surrounding the principal sulcus. The brains were sectioned coronally at 60 μm on a vibratome (Leica) across the entire rostrocaudal extent of the dorsolateral prefrontal cortex (dlPFC; Walker’s area 46). The sections were cryoprotected through increasing concentrations of sucrose solution (10%, 20% and 30% each for 2 hr, then 30% overnight), cooled rapidly using liquid nitrogen and stored at -80°C. Sections of dlPFC were processed for immunocytochemistry. To enable penetration of immunoreagents, all sections went through 3 freeze-thaw cycles in liquid nitrogen.

**Lysis comparison procedures**

For comparison, in **Figure 1C,D** all lysates were made at a ratio of 4 μl of the respective lysis buffer for every 1 mg of tissue to start, except for 1% triton-soluble samples which were prepared as described (Datta et al., 2021). Urea samples were lysed in 8 M urea 0.4 M ammonium bicarbonate via sonication. Non-soluble debris was cleared with a 16,000 x g 10 min centrifugation. “1% SDS + heat” samples were prepared in a 1% SDS lysis buffer with phosphatase and protease inhibitor. Samples were first sonicated, then boiled for 5 min at 100°C, and supernatant was collected following 5 min 16,000 x g centrifugation. Each well of the gel was loaded with an appropriate volume of lysates such that the lanes are representative of the same amount (mg) of starting tissue, the exact volume for each is listed in the figure legend **Figure 1C,D.**

**LC-MS/MS on the Thermo Scientific Q Exactive Plus**

Peptides were dissolved in 25 µl MS loading buffer (2% ACN, 0.2% TFA), with 5 µl injected for LC-MS/MS analysis. LC-MS/MS analysis was performed on a Thermo Scientific Q Exactive Plus equipped with a Waters nanoAcquity UPLC system utilizing a binary solvent system (A: 100% water, 0.1% formic acid; B: 100% acetonitrile, 0.1% formic acid). Trapping was performed at 5 µl/min, 99.5% Buffer A for 3 min using a Waters nanoACQUITY UPLC M-Class Symmetry C18 Trap Column (100Å, 5 µm, 180 µm x 20 mm, 2G, V/M). Peptides were separated at 37°C using a Waters nanoACQUITY UPLC Peptide BEH C18 Column (130Å, 1.7 µm, 75 µm x 250 mm) and eluted at 300 nl/min with the following gradient: 3% buffer B at initial conditions; 5% B at 2 min; 25% B at 140 min; 40% B at 165 min; 90% B at 170 min; 90% B at 180 min; return to initial conditions at 182 min.  MS was acquired in profile mode over the 300-1,700 m/z range using 1 microscan, 70,000 resolution, AGC target of 3E6, and a maximum injection time of 45 msec.  Data dependent MS/MS were acquired in centroid mode on the top 20 precursors per MS scan using 1 microscan, 17,500 resolution, AGC target of 1E5, maximum injection time of 100 msec, and an isolation window of 1.7 m/z.  Precursors were fragmented by HCD activation with a collision energy of 28%.  MS/MS were collected on species with an intensity threshold of 1E4, charge states 2-6, and peptide match preferred.  Dynamic exclusion was set to 20 sec. Chemicals, including water, used for mass spectrometry were purchased in glass bottles to prevent plastic contamination. Products are available through Sigma or Millipore.

**Western blot procedures**

Samples were run on 4-20% tris-glycine gels and transferred onto 0.2 μm nitrocellulose membranes. Membranes were blocked with 5% milk and incubated overnight with primary antibody at 4°C. Blots were developed on a LI-COR odyssey scanner using fluorescent secondary antibodies for the appropriate species. Quantification was done in ImageStudio Lite where background was subtracted by calculating the average intensity immediately above and below the band(s) of interest. All protein levels were normalized to GAPDH for quantification. Statistical analyses were performed in Graphpad Prism. The normalcy of the protein value distribution was tested using a D’Agostino and Pearson normality test. The appropriate test was chosen on the basis of these results and is listed in the figure legend for either a comparison of means or correlation with age.

**Data Analysis**

Spectra were searched on Mascot version 2.7.0 in Proteome Discoverer 2.2.0.388 with a parent tolerance of 10 ppm (monoisotopic), fragment tolerance of 0.020 Da (monoisotopic), and strict trypsin digestion with a maximum of 3 missed cleavages. Variable modifications included oxidation (M), carbamidomethylation (C), phosphorylation (S, T, Y), acetylation (K, protein N-terminus), GG (K)(ubiquitin remnant), deamidation (N, Q), and DeStreak (C)(beta-mercaptoethanol adduct). Samples were searched with a species-specific general protein library as well as a 2N4R tau-specific library. A 0.3% peptide false discovery rate (FDR) and 7.0% protein FDR were used for macaque results and a 0.4% protein FDR was the only threshold for rodent results as listed in the supplementary tables.

Scaffold PTM re-analyzes MS/MS spectra identified as modified peptides and calculates Ascore values and site localization probabilities to assess the level of confidence in each PTM localization. Scaffold PTM then combines localization probabilities for all peptides containing each identified PTM site to obtain the best estimated probability that a PTM is present at that particular site. All tau phosphosites identified in this study had a localization probability ≥96%.

Total Ion Current (TIC) analysis was compared to spectral count analysis to ensure that an accurate representation of the data was presented. TIC was obtained from Scaffold for total 2N4R tau and TIC for each phosphosite was obtained from Scaffold PTM. TIC values represent the “sum of all the TIC values of all spectra assigned to a protein” and TIC is “the sum of the areas under all the peaks contained in a MS/MS spectrum”. TIC values were calculated with a minimum value of 0.0. For modified sites TIC sums the TIC values of all spectra representing the modification site.

The correlation matrix was created using R Studio cor and corrplot. The correlation coefficient is used to determine the color and size of the cell for each correlation. No statistical significance was assigned given the small sample size for macaque proteomics data presented in the manuscript.

**Supplemental Figures**

**
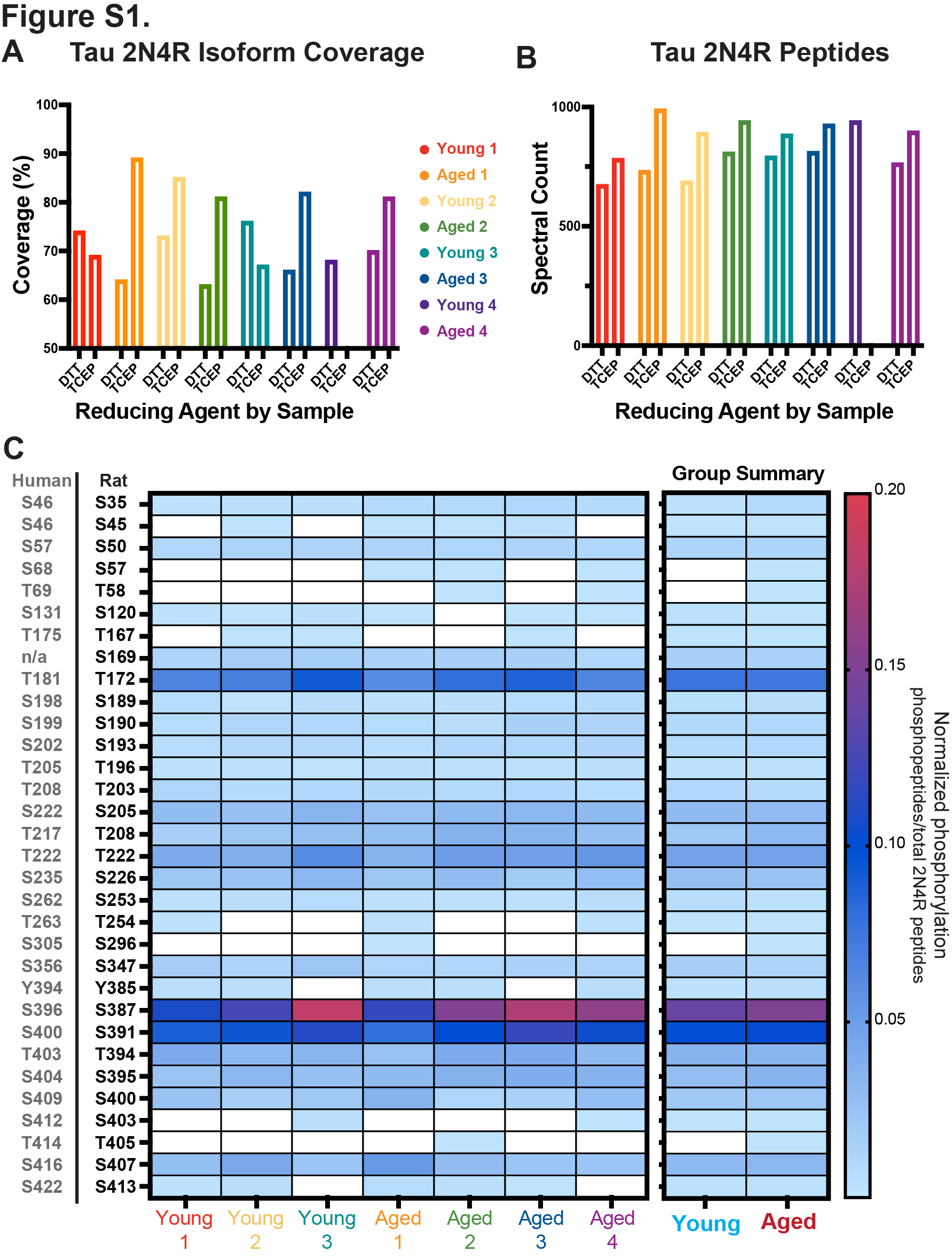
**

**Figure S1. Mass spectrometry analysis of age-related changes in rat tau phosphorylation**

A) Bar graph representation of the percent coverage of the 2N4R isoform of rat tau achieved in each sample run. All samples were TiO_2_ enriched. Results are color coded by the animal from which the sample was derived and are plotted for both DTT and TCEP reductions. B) Bar graph representation of the 2N4R tau spectral counts detected for each of the samples presented in panel A. C) Heat map representation of the phosphoproteomic results from TCEP reduced, TiO_2_ enriched, analysis of young (3.5 months of age, N=3) and aged (30 months of age, N=4). Results are presented as the normalized spectral count calculated as the spectral count of phosphopeptides encompassing a given residue over the total spectral counts of 2N4R tau. Individual samples are plotted on the left and a group summary with the mean value for each residue by group is presented on the right. White cells indicate there were no identified phosphopeptides for that residue in the sample(s). On the left-hand side of the heat map, residues are labelled by the position of the phosphorylation site according to 2N4R rat tau numbering and the analogous human site is identified at the farthest left in grey. A 2-way ANOVA was run for the groupwise comparison. The individual residue analyzed was a significant source of variation (**** p<0.0001) as was the individual animal (*p=0.0147) but there was no significant effect of age or phosphosite.


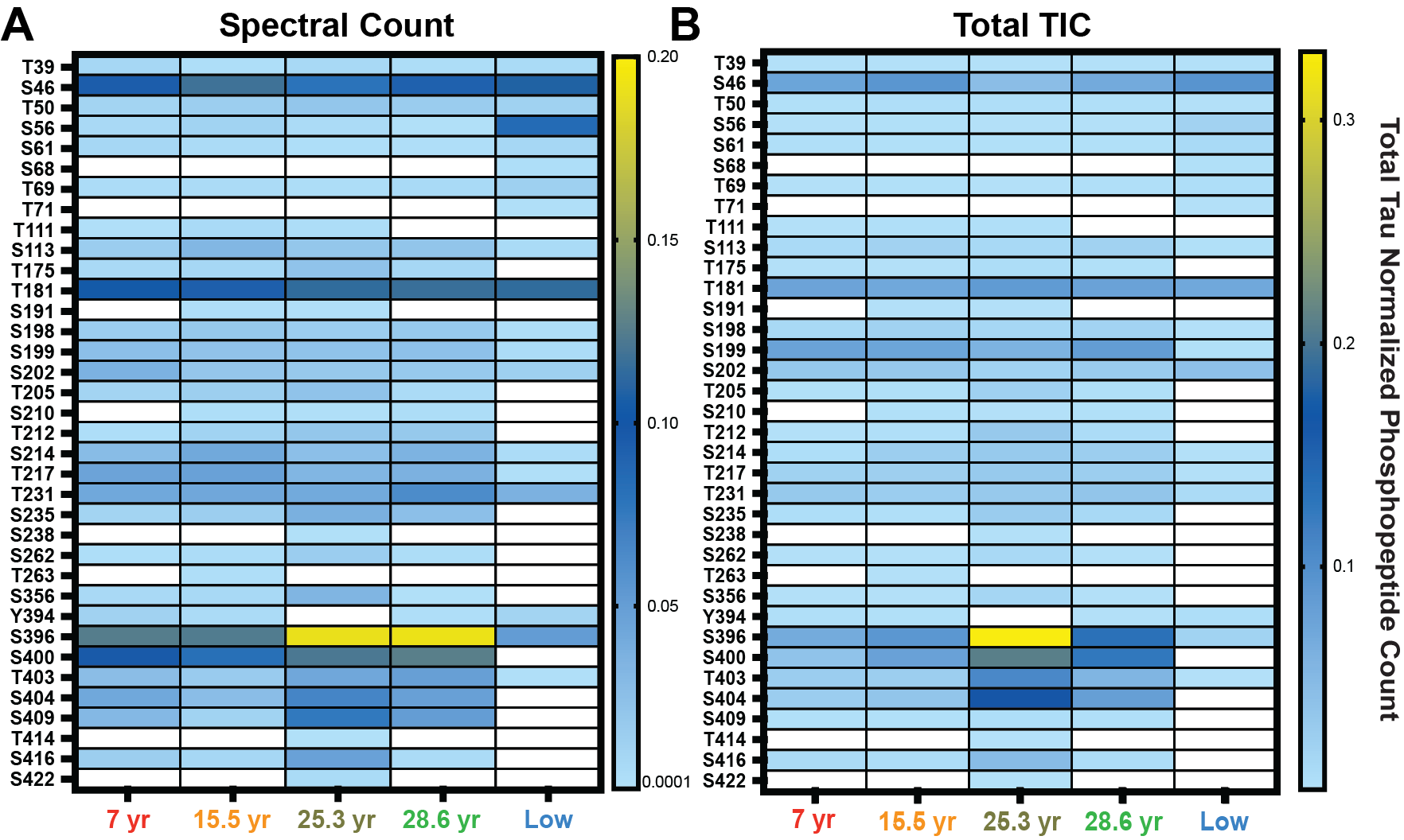


**Figure S2. Comparison of Spectral Count and Total TIC quantification**

A) The same heat map as Figure 2E representing the normalized phosphorylation value at each site for all animals via normalized spectral count (phosphorylated spectral count/ total tau spectral count). B) A heat map with the same characteristics as A but utilizing values computed using total TIC instead of spectral count.


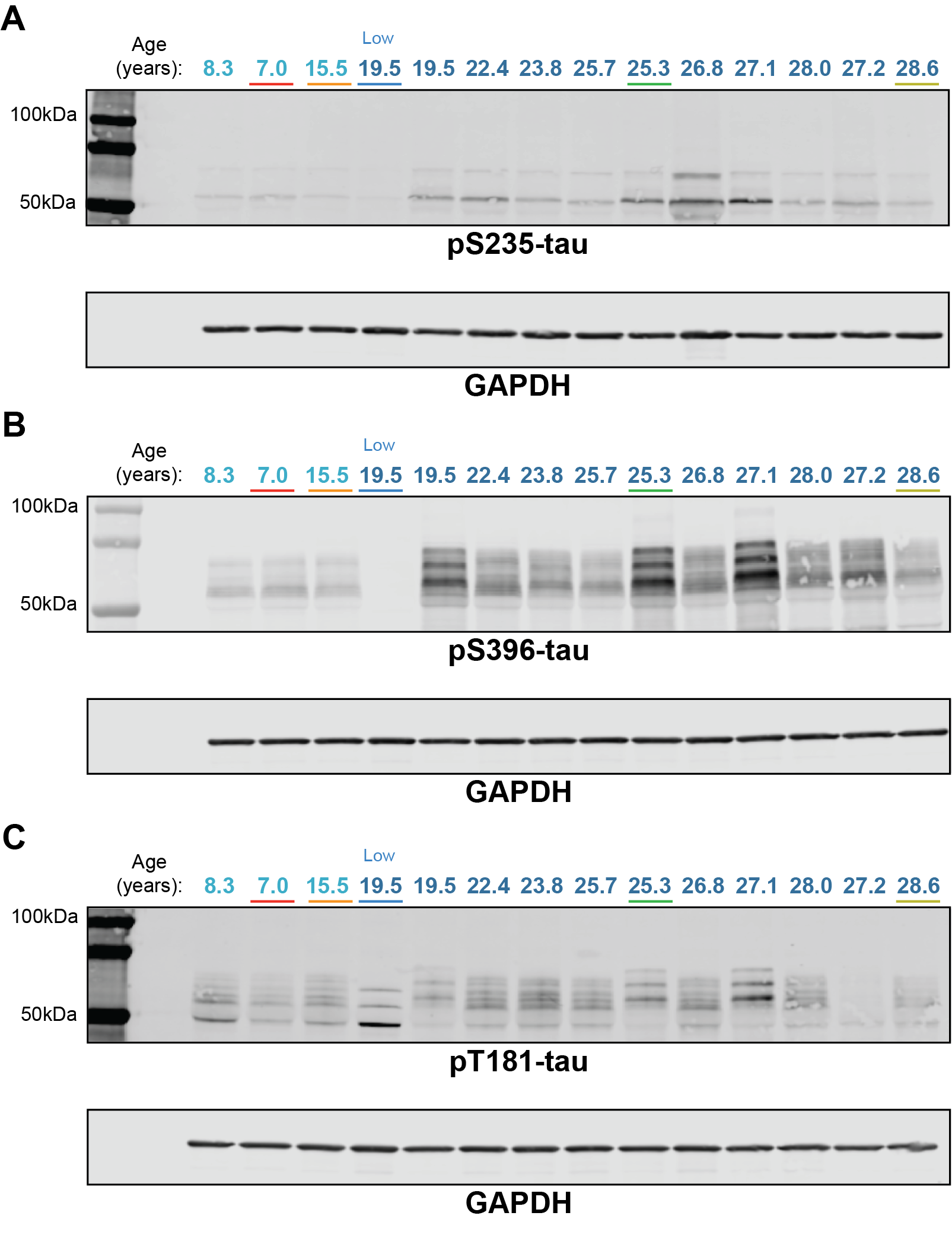


**Figure S3. Immunoblots of pT181-tau, pS235-tau and pS396-tau**

Animals are labelled by their age in years and animals that have phosphoproteomic data are underlined and color coded to match their identification in Figure 2. A) Immunoblot of pS235-tau and GAPDH. Each lane contained 20 μg from an individual macaque 1% triton-soluble lysate. B) Immunoblot of the same lysate on a separate membrane probed with pS396-tau and GAPDH antibodies. C) Immunoblot of the same lysate on a separate membrane run on a different day probed with pT181-tau and GAPDH antibodies.

**
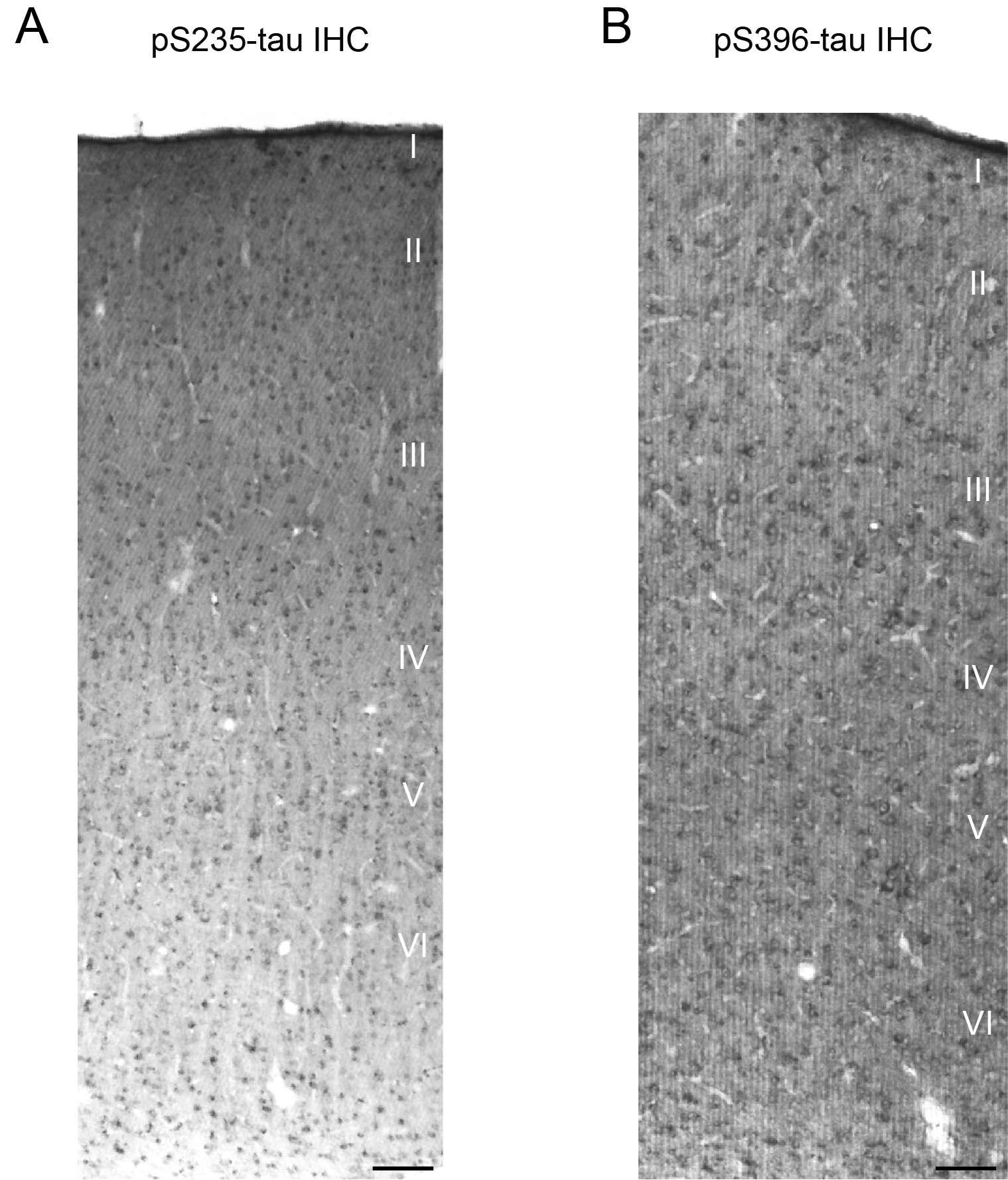
**

**Figure S4. pS235-tau and pS396-tau immunohistochemistry across cortical layers in aged macaque dlPFC.**

A) Immunolabeling for pS235-tau in aged macaque dlPFC. Immunopositive cells are distributed throughout the cortical neuropil across all layers. Scale bar: 100 µm. B) Immunolabeling for pS396-tau in aged macaque dlPFC showing labeled cells across all cortical layers. Approximate depth of cortical layers is demarcated in each micrograph. Scale bar: 80 µm.

**Supplemental Tables**

**Table S1. Rat phosphoproteomics data**

Tab 1: Output of spectral counts from all protein data from Scaffold

Tab 2: Quantification of normalized phosphorylation by spectral count incorporating Scaffold protein data and Scaffold PTM phosphorylation data.

**Table S2. Rat phosphoproteomics data**

Tab 1: Output of spectral counts from all protein data from Scaffold. Tau 2N4R is highlighted in yellow. The color-coded key of animal IDs is included at the top as are the details of the search.

Tab 2: Tau 2N4R phosphopeptide data from Scaffold PTM is presented on the right. Sum of phosphopeptide spectral counts by region is on the top left. Percentage of total phosphopeptides by region is on the bottom left.

Tab 3: Quantification of normalized phosphorylation by spectral count incorporating Scaffold protein data and Scaffold PTM phosphorylation data

Tab 4: Quantification of normalized phosphorylation by total TIC values incorporating Scaffold protein data and Scaffold PTM phosphorylation data. The total TIC output from scaffold are in the outlined cells at the top of the sheet and the normalized values with the total TIC output from Scaffold PTM are below.

**Works Cited**

Bartus, R.T., Fleming, D., and Johnson, H.R. (1978). Aging in the rhesus monkey: debilitating effects on short-term memory. *J Gerontol* 33**,** 858-871.

Datta, D., Leslie, S.N., Wang, M., Morozov, Y.M., Yang, S., Mentone, S., Zeiss, C., Duque, A., Rakic, P., Horvath, T.L., Van Dyck, C.H., Nairn, A.C., and Arnsten, A.F.T. (2021). Age-related calcium dysregulation linked with tau pathology and impaired cognition in non-human primates. *Alzheimers Dement*.

Leslie, S.N., Datta, D., Christensen, K.R., Van Dyck, C.H., Arnsten, A.F.T., and Nairn, A.C. (2020). Phosphodiesterase PDE4D Is Decreased in Frontal Cortex of Aged Rats and Positively Correlated With Working Memory Performance and Inversely Correlated With PKA Phosphorylation of Tau. *Front Aging Neurosci* 12**,** 576723.
